# Editorial: Role of Ribonucleases in Immune Response Regulation During Infection and Cancer

**DOI:** 10.3389/fimmu.2020.00236

**Published:** 2020-02-19

**Authors:** Ester Boix, Francesco Acquati, Demetres Leonidas, David Pulido

**Affiliations:** ^1^Department of Biochemistry and Molecular Biology, Faculty of Biosciences, Universitat Autònoma de Barcelona, Cerdanyola del Vallès, Spain; ^2^Human Genetics Laboratory, Department of Biotechnology and Molecular Sciences, University of Insubria, Varese, Italy; ^3^Department of Biochemistry and Biotechnology, University of Thessaly, Biopolis, Larissa, Greece; ^4^The Jenner Institute, Medical Sciences Division, Nuffield Department of Medicine, University of Oxford, Oxford, United Kingdom

**Keywords:** RNaseA, RNaseT2, cancer, infection, innate immunity, oligomers

Ribonucleases (RNases) are key players of the host immunity and contribute to maintaining tissue homeostasis and body fluid sterility. Secreted upon a diversity of cellular injuries, they mediate signaling processes, and have been classified as alarmins (1). Recent literature reveals how the immune response system uses common strategies to fight both cancer and infection. RNases participate in immune system adaptation to cellular stress conditions. They can shape the metabolism of cellular RNA, specifically target the non-coding RNA population, or even induce signal transduction in a catalytically independent manner (2–4). This Research Topic gathers some of the latest research on two secretory RNase families, RNaseA and RNaseT2, which participate in the host immune response and share similar mechanisms of action.

The RNaseA family takes its name from bovine pancreatic RNaseA, one of the first and best enzymes characterized in the early 20th century (5, 6). It is vertebrate-specific and includes eight functional members in humans (7). In turn, the RNaseT2 family is broadly distributed across all kingdoms and has a unique member in humans (8). Nonetheless, we find huge similarities in their catalytic mechanisms and biological properties. Interestingly, both proteins have catalytically dependent and independent immune-regulatory activities. In this issue, we include reviews and original research articles that focus on the immune defense role of the RNases in infection and cancer.

Within the RNaseA family, we find several members with antimicrobial properties (2). Here, Boix et al. identified the human canonical members that mediate the eradication of macrophage infection. Interestingly, the removal of intracellular dwelling bacteria is not dependent on the protein catalytic activity and is mediated by autophagy induction. The authors also observed how bacterial infection modulates the expression of endogenous RNases 3 and 6, suggesting a physiological role. In turn, the review by Spencer and co-workers highlights the enhanced expression of both RNases in urinary tract infection. The RNases participate together with other antimicrobial peptides in the bacterial clearance at the uroepithelial barrier. The authors propose a task specialization whereby RNases 3 and 6 are released by activated infiltrating eosinophils and macrophages, respectively, and two additional RNases, RNases 4 and 7, are constitutively produced by epithelial cells to reinforce the kidney and bladder protection. In addition, RNase7 is abundantly released by keratinocytes and can protect the skin from pathogens. Harder et al. explore the role of skin RNase7 during infection, with special attention to its immunomodulatory activities. The review includes the outlook of their very recent work, showing how RNase7 senses the host self-DNA as a skin damage signal and the subsequent activation of the cell antiviral response.

On the other hand, secretory RNases are also protecting our tissues against other cell injuries, such as cell cycle dysregulation in tumorigenesis. Indeed, RNases have long been known to be involved in the control of cancer growth (9). Several members of the RNaseA family have been reported to carry out oncosuppressive activity and have been proposed for antitumor therapy (10). Within this context, the review by Gotte and Menegazzi explores the potential of RNaseA members to act as antitumor drugs. Secreted RNases can be cytotoxic when entering the cell. Fortunately, the vertebrate cell cytosol has a major protective shield against RNases, the proteinaceous ribonucleolytic inhibitor (RI). One way to evade RI is the intrinsic ability of RNases to oligomerize and form homo- and even heterodimers. The activities of RNase natural and artificial oligomers are analyzed in light of the development of RNase-based therapeutic applications.

Strikingly, RNases belonging to the T2 protein family have also recently been implicated in tumor suppression (8). Specifically, the human RNASET2 gene has been consistently reported to act as a powerful, pleiotropic tumor suppressor (11). Of note, tumor suppression turned out to be an evolutionarily conserved feature of T2 RNases (12) and to be carried out by means of both cell-autonomous and non-autonomous mechanisms, among which the establishment of cancer cell-tumor microenvironment cross-talk was established to play a key role (13). In fact, recent studies have defined key cellular components of the innate immune system, such as macrophages, as critical effectors of RNASET2-mediated tumor suppression in both *in vitro* and *in vivo* experimental models (14).

Within the frame of this Research Topic, the review by Acquati et al. summarizes the role played by members of the very ancient ribonuclease T2 gene family in innate immunity-mediated tumor suppression. The review provides an overview of recent data demonstrating an evolutionarily conserved role for T2 RNases in the recruitment and/or activation of key innate immunity effector cells in several experimental models. Noteworthily, in keeping with the growing bulk of experimental evidence pointing at the innate immune system as a key effector in cancer growth control, the recently established role of T2 RNases in the regulation of innate immunity seems to represent a key component of their tumor-suppressive activity. Indeed, *in vivo* studies focused on the only human member of the T2 RNase family have consistently shown that its tumor-suppressive activity is strictly dependent on the ability to recruit antitumor, M1-polarized host macrophages. Moreover, the reported role of T2 RNases as wide-range stress response genes prompted the authors to include RNASET2 in the growing family of alarmins, as molecules passively released by necrotic cells or actively secreted by epithelial or immune cells to alert of dangerous events, such as the local occurrence of early-stage cancers.

Despite the extensive work applied to unraveling the mechanism of action of secretory RNases, little is known about their physiological RNA substrates and how the released RNA products regulate host immunity. Interestingly, recent reports reveal the key role of non-coding RNA fragments, such as tRNA halves, that are generated upon the cleavage of specific RNases and are associated with signaling pathways (15). Thereby, secretory RNases will contribute to enlarge the ncRNA diversity and functionality (16). Mastering their cleavage selectivity and immune-modulation properties should provide us the novel tools for the specific removal of dysfunctional cells and the maintenance of healthy tissues. We are confident that a better understanding of the role of RNases would contribute to the development of new therapies against both cancer and infection.

## Author Contributions

EB, FA, DL, and DP participated in the first manuscript draft. EB edited the final version and all authors revised it. All authors listed have made a substantial, direct and intellectual contribution to the work, and approved it for publication.

### Conflict of Interest

The authors declare that the research was conducted in the absence of any commercial or financial relationships that could be construed as a potential conflict of interest.
